# Complete and Draft Genome Sequences of Aerobic Methanotrophs Isolated from a Riparian Wetland

**DOI:** 10.1128/MRA.01438-20

**Published:** 2021-03-04

**Authors:** Ohana Yonara de Assis Costa, Marion Meima-Franke, Paul L. E. Bodelier

**Affiliations:** a Department of Microbial Ecology, Netherlands Institute of Ecology (NIOO-KNAW), Wageningen, The Netherlands; University of Southern California

## Abstract

Wetlands are important sources of methane emissions, and the impacts of these emissions can be mitigated by methanotrophic bacteria. The genomes of methanotrophs *Methylomonas* sp. strain LL1 and *Methylosinus* sp. strain H3A, as well as *Methylocystis* sp. strains H4A, H15, H62, and L43, were sequenced and are reported here.

## ANNOUNCEMENT

Wetlands are among the largest source of the greenhouse gas methane (1). Methanotrophic bacteria are the only biological filter, mitigating emission to the atmosphere (1). Here, we report the complete genome sequence of *Methylomonas* sp. strain LL1 and the draft genome sequences of *Methylosinus* sp. strain H3A and *Methylocystis* sp. strains H4A, H15, H62, and L43. These bacteria were isolated as previously described (2) from the same riparian wetland where composition and functioning of the methanotrophic community were described (3–5).

Strains were grown as described previously (2) in a nitrate mineral salts (NMS) medium, at 25°C in an atmosphere of 30% CH_4_ in air, and were harvested in mid-exponential phase. Bacterial genomic DNA was extracted using either the GNOME DNA isolation kit (MP Biomedicals, USA) (all *Methylocystis* sp. strains) or the Genomic-tip 100/G kit (Qiagen Benelux BV, The Netherlands) (*Methylomonas* sp. strain LL1 and *Methylosinus* sp. strain H3A), according to the manufacturers' instructions. The sequencing libraries of the genomes of *Methylomonas* sp. strain LL1 and *Methylosinus* sp. strain H3A were prepared using the SMRTbell template prep kit 1.0 and sequenced in one single-molecule real-time (SMRT) cell using the PacBio RS II (Pacific Biosciences, Inc., USA) sequencing platform, which was executed by the Genomics Facility of the School of Medicine of the University of Maryland (Baltimore, USA). Quality control, raw read filtering, and genome assembly were performed with the help of SMRT analysis software v2.3.0 (Pacific Biosciences, Inc.) featuring Hierarchical Genome Assembly Process algorithm v3 (HGAP3) (6). The HGAP3 data processing pipeline comprised PreAssembler v1 for filtering, Celera assembler v8.1 for assembly (7), BLASR v1 (8) for mapping, and Quiver v1 (6) for consensus polishing using only unambiguously mapped reads. HGAP3 defaults settings were applied, except for the genome size estimate parameter, set to 5.0 Mbp. The completeness of the *Methylomonas* sp. strain LL1 genome was assessed using the benchmarking universal single-copy orthologs (BUSCO; v4.14) software (https://busco.ezlab.org) (9) with the gammaproteobacteria_odb10 database (quality score, 98.9%; 366 total BUSCO groups searched). The genomes of all *Methylocystis* sp. strains were sequenced using an Illumina HiSeq 2000 instrument. Library preparation (10), sequencing, and sequence quality control and trimming using internally developed software SOAPnuke v1.4.0 and parameters -l 15 -q 0.2 -n 0.05 (11) were performed at BGI Tech Solutions (Hong Kong, China). The sequencing library was prepared according to BGI protocols. Briefly, 1 μg genomic DNA was randomly fragmented by a Covaris sonicator. DNA fragments were end repaired and A tailed. Next, Illumina adapters were ligated to the 3′-adenylated DNA, which was purified using AxyPrep Mag PCR clean up kit. The resulting fragments were size selected by agarose gel electrophoresis. The library quality and quantity were assessed using the Agilent Technologies 2100 bioanalyzer and ABI StepOnePlus real-time PCR system. The quality of all reads was checked by FastQC v0.11.9 (http://www.bioinformatics.babraham.ac.uk/projects/fastqc/). Clean reads were assembled using Ray v2.0.1 (12). Automatic gene prediction and annotation were performed by using NCBI Prokaryotic Genome Annotation Pipeline (PGAP) (13). Default parameters were used for all software unless otherwise specified. The *Methylomonas* sp. LL1 genome includes a 0.12-Mb plasmid. General genome statistics and predicted metabolic pathways are detailed in Table 1.

**TABLE 1:** General genome information

Strain	Sequencing platform	Total size of assembly (Mb)	Assembler	No. of raw reads	Raw read *N*_50_ (bp)	Coverage (×)	Completion	G+C content (%)	No of CDS^*a*^	No of scaffolds	Scaffold *N*_50_ (bp)	Core metabolic pathways^*b*^	GenBank (SRA) accession no.^*c*^
*Methylomonas* sp. LL1	PacBio RS II	4,923,893	Celera v8.1	78,774	14,377	16	Yes	50.9	4,426	2	4,798,577	pMMO, Mox, PQQ, sMMO, SC, PPP, RuMP, EDD, EMP, TCA	CP064653.1, CP064652.1 (SRR13259185)
*Methylosinus* sp. H3A	PacBio RS II	5,384,582	Celera v8.1	63,638	15,418	16	No	64.2	4,974	11	4,391,449	pMMO, Mox, PQQ, sMMO, SC, EMP, TCA	JADNQW000000000 (SRR13259182)
*Methylocystis* sp. H15	Illumina HiSeq 2000	4,053,493	Ray 2.0.1	6,891,600		49.8	No	62.3	3,913	39	217,286	pMMO, Mox, PQQ, sMMO, SC, EMP, TCA	JADNQX000000000 (SRR13259184)
*Methylocystis* sp. H4A	Illumina HiSeq 2000	4,288,666	Ray 2.0.1	6,990,370		49.8	No	62.3	4,214	38	238,602	pMMO, Mox, PQQ, sMMO, SC, EDD, EMP, TCA	JADNQY000000000 (SRR13259180)
*Methylocystis* sp. H62	Illumina HiSeq 2000	4,540,521	Ray 2.0.1	6,914,734		43.9	No	62.1	4,408	63	216,123	pMMO, Mox, PQQ, sMMO, SC, EMP, TCA	JADNQZ000000000 (SRR13259181)
*Methylocystis* sp. L43	Illumina HiSeq 2000	4,061,225	Ray 2.0.1	6,899,554		48.6	No	62.4	3,919	38	145,473	pMMO, Mox, PQQ, sMMO, SC, EMP, TCA	JADNRA000000000 (SRR13259183)

a CDS, coding DNA sequences.

b pMMO, membrane-bound methane monooxygenase; Mox, PQQ-linked methanol and formaldehyde dehydrogenases; PQQ, pyrroloquinoline quinone biosynthesis; sMMO, soluble methane monooxygenase; SC, serine cycle; PPP, pentose phosphate pathway; RuMP, assimilatory ribulose monophosphate pathway; EDD, Entner-Doudoroff pathway; EMP, Embden-Meyerhof-Parnas pathway; TCA, tricarboxylic acid cycle.

c The BioProject number of the study is PRJNA674997.

### Data availability.

GenBank and SRA (raw data) accession numbers are listed in Table 1.
